# The pediatric rheumatology quality of life scale: validation of the English version in a US cohort of juvenile idiopathic arthritis

**DOI:** 10.1186/1546-0096-11-43

**Published:** 2013-11-08

**Authors:** Pamela F Weiss, Andrew J Klink, Jennifer Faerber, Chris Feudtner

**Affiliations:** 1 Division of Rheumatology, The Children’s Hospital of Philadelphia, Room 1526, North Campus, 3535 Market Street, Philadelphia, PA, USA; 2 Division of General Pediatrics, The Children’s Hospital of Philadelphia, Philadelphia, PA, USA; 3 Center for Pediatric Clinical Effectiveness, The Children’s Hospital of Philadelphia, Philadelphia, PA, USA; 4 Department of Pediatrics, Center for Clinical Epidemiology and Biostatistics, University of Pennsylvania Perelman School of Medicine, Philadelphia, PA, USA; 5 Leonard Davis Institute of Health Economics, University of Pennsylvania Perelman School of Medicine, Philadelphia, PA, USA

**Keywords:** Quality of life, Patient-reported outcomes, Pediatric, Juvenile arthritis

## Abstract

**Background:**

This study aims to validate the English version of the Pediatric Rheumatology Quality of Life Scale (PRQL), a concise Health Related Quality of Life (HRQoL) measure, in a US cohort of children with juvenile idiopathic arthritis (JIA).

**Methods:**

The PRQL is a 10-item HRQoL measure with two subscales: physical health and psychological health. The original version of this measure was validated using an Italian-speaking cohort of 472 JIA patients and 796 healthy controls and found to have acceptable psychometric properties. The English language version has not been validated in a US pediatric population. The English PRQL was administered to 161 JIA subjects from a US Rheumatology clinic. We assessed the reliability (internal consistency and test-retest) and validity (convergent, discriminative, and criterion) of the PRQL.

**Results:**

The English PRQL was feasible to administer and demonstrated good psychometric properties. Cronbach alpha (reliability) coefficients ranged from 0.72 to 0.81. Factor analysis yielded the existing subscales. The PRQL total and subscales were found to have moderate correlations with other HRQoL instruments, the Pediatric Quality of Life Inventory (PedsQL) generic core scale and the PedsQL rheumatology. The PRQL discriminated between subjects with active versus inactive disease and was responsive to an improvement or worsening in disease activity over time.

**Conclusions:**

Our results suggest that the English version of the instrument is suitable for use in JIA patients in the US. This tool provides a relatively easy method to integrate at least one patient-reported outcome into routine clinical or research assessment.

## Background

The importance of patient-reported outcomes (PROs), reported by pediatric patients and their parents, is increasingly emphasized in both routine clinic care and research. PROs include items such as health related quality of life (HRQoL), functional status, pain, and satisfaction. Recently, the Consolidated Standards of Reporting Trials (CONSORT) patient reported outcome extension was released that urged inclusion of PROs as primary or secondary outcomes in ancillary analyses for all randomized controlled trials [1]. PROs are also important for high-quality patient centered care and comprehensive decision-making. HRQoL is a particularly important PRO to include in the overall assessment of children with juvenile idiopathic arthritis (JIA). If clinical assessment is limited to physician-determined items such as active joint count, the impact of factors such as medication side effects, fear of injections, and missed school for medication infusions may be overlooked.

Several validated HRQoL measures exist to measure HRQoL in children with rheumatology conditions e.g. the Pediatric Quality of Life Inventory (PedsQL) and the Juvenile Arthritis Quality of Life Questionnaire (JAQQ)), but these existing instruments are either time consuming, costly, and/or complicated to administer in clinical settings. The PedsQL has been validated and used extensively in a wide range of diseases, including JIA [2-6]. However, the PedsQL Generic Core contains 23 items and the PedsQL Rheumatology Module contains 22 items, rendering them cumbersome to complete during a clinic visit. Further, the PedsQL Generic Core and Rheumatology modules are costly to use in routine clinical practice [7]. The JAQQ is a validated rheumatology specific measure that has also been used in JIA and outcomes studies [8-11]. Similar to the PedsQL it is time consuming to complete with 74 items. The ability of children to select the 5 most problematic areas in each domain to answer allows for a unique individualized assessment over time but makes comparison of scores across children problematic [12].

The feasibility constraints of the previous HRQoL instruments led to the development of the Pediatric Rheumatology Quality of Life Scale (PRQL), a concise 10-item HRQoL measure designed for use in the clinical setting but with potential utility for research [13]. Items included in the PRQL were derived from literature review, analysis of existing HRQoL measures, study panel discussion, and face-to-face interviews of children with rheumatic disease and their parents. Two independent individuals translated the Italian version of the PRQL to English [14]. Two native-English translators then independently translated the English version back to Italian. The translation was assessed by a focus group of Pediatric Rheumatology International Trials Organization (PRINTO) employees with translation expertise to ensure the content of the instrument was preserved across translation. The Italian version of PRQL was validated using an Italian cohort of 472 JIA patients and 796 healthy controls. The median age of JIA patients was 8.7 (IQR: 4.7, 12.2) years. The comparison group of healthy children had a similar age distribution [13]. The PRQL possessed good measurement properties, including feasibility and face, content, and construct validity, test-retest reliability, and good responsiveness to patient improvement over time. A Cronbach’s α of 0.86 demonstrated good internal consistency. The PRQL is available in English but has not been validated in a US pediatric population [13].

The PRQL is an attractive option for use in routine clinical practice because it is free, takes less than 5 minutes to complete, and less than 1 minute to score. The aim of this study was to validate the English PRQL in JIA subjects from our US clinical practice, using the PedsQL (generic core and rheumatology module) as the gold standard.

## Methods

### Human subjects protections

The protocol for the conduct of this study was reviewed and approved by the Committee for the Protection of Human Subjects.

### Subjects

The source population for the study was children with a diagnosis of JIA who were 18 years of age or younger and evaluated in the rheumatology clinic between August 2011 and June 2012. Subjects were a convenience sample enrolled without regard to disease duration, disease severity, current disease activity, or therapy. All subjects met International League of Associations for Rheumatology (ILAR) criteria [15] for JIA according to the treating physician. 96% (161/167) of people approached agreed to participate in the study.

### Clinical data

The following demographics and clinical characteristics were collected at the study visit: age, disease duration, sex, race, and ILAR category.

### Measures

#### Quality of life

The PRQL, PedsQL generic scale (version 4.0), and the PedsQL-Rheumatology module (version 3.0) were completed by the parent(s)/legal guardian(s) or by the participant if 13 to 18 years old.

The PRQL is a concise 10-item QOL measurement that uses 4-point Likert scales from 0 (never) to 3 (all the time). Items are grouped into 2 subscales, physical health (PhH) and psychosocial health (PsH). The total score ranges from 0 to 30, where higher scores indicate worse HRQoL. If more than 2 questions are not answered in each sub-domain, the PRQL score is not computed [13].

The PedsQL has been validated and used extensively in a wide range of diseases, including JIA [2-6]. The PedsQL has a validated rheumatology module for use among patients with JIA [6]. The PedsQL Generic Core contains 23 items, and the PedsQL Rheumatology Module contains 22 items. Both the generic and rheumatology questionnaires use 5-point Likert scales from 0 (never) to 4 (almost always). Scores are transformed on a scale from 0 to 100, where higher scores indicate better HRQoL.

#### Pain intensity

At enrollment the subject or parent was asked, “How much pain have you [or your child] had because of your [his/her] rheumatic condition in the past week?” Pain intensity was reported using integers from 0 to 10, anchored by the words “No Pain” and “Very Severe Pain”. High correlation between the traditional 10-centimeter visual analogue scale (VAS) and the integer-based scale has been reported for the measurement of pain intensity [16-18].

#### Physical function

Subjects or parents rated the subject’s disease-related functional status during the past week using the Childhood Health Assessment Questionnaire (CHAQ) [19]. A disability index (DI) is calculated based on the mean of the 8 domains; the DI ranges from 0 to 3, with higher scores indicating worse disability [20].

#### Parent/subject global health status

At enrollment the subject or parent was asked, “Considering all the ways that arthritis affects you/your child, how do you rate how he/she is doing in the past week?” Status was reported using integers from 0 to 10, anchored by the words “Very well” and “Very poor”.

#### Disease activity

A physician disease activity VAS was reported for each visit using integers from 0 to 10. Physicians also determined whether subjects met criteria for inactive disease according to the ACR provisional criteria [21]. Subjects who did not fulfill these criteria were considered as having active disease.

#### Data collection

Study data were managed using Research Electronic Data Capture (REDCap) tools hosted at Children’s Hospital of Philadelphia [22].

#### Procedure

In order to assess test-retest reliability, a small subset of subjects were asked to retake the PRQL. Forty-three enrolled subjects or parents were emailed a copy of the PRQL one week after the initial administration of the questionnaire. A period of one week was used, as it is not too short that respondents will not remember their first response and not too long that the patients’ HRQoL will have changed [23].

### Analysis

We assessed the validity of the English version of the PRQL using the Outcome Measures in Rheumatoid Arthritis Clinical Trials (OMERACT) filter [24]. The OMERACT filter was developed to simplify and standardize the terminology used in rheumatology outcome and validation studies.

#### Internal consistency reliability

Internal consistency was determined using Cronbach’s alpha coefficient. Estimates greater than or equal to 0.70 were considered acceptable.

#### Test-retest reliability

Test-retest reliability was assessed using the intraclass correlation coefficient (ICC) for total and both subscale scores.

#### Confirmatory factor analysis

Factor analysis is used to identify over-arching factors that account for the common variance in the observed PRQL items, excluding item-specific (unique) variance [25]. We used confirmatory factor analysis (CFA which is a type of factor analysis,) to test how well the 2-factor model obtained for the Italian version of the instrument fit our data. CFA was conducted since this instrument’s factor structure is already known from previous research. A Bentler’s Comparative Fit Index (CFI) > 0.9, Bentler & Bonnett’s Non-Normed Fit Index (NNI) > 0.9, and the root mean square error of approximation (RMSEA) < 0.1 were considered indicative of an acceptable model fit [26].

#### Post-hoc exploratory factor analysis

A post-hoc exploratory factor analysis of the PRQL was also performed to check if the two-factor model fit would be confirmed without making assumptions about the data. We performed a common factor analysis, which is more suitable for identifying latent factors than principal components analysis. A maximum likelihood factor analysis was performed in which squared multiple correlations were used for the initial commonality estimates. A promax (oblique) rotation was used to identify the underlying factor structure.

#### Convergent and discriminative validity

Convergent validity of the candidate index was tested using the PedsQL 4.0 generic scale integrated with the PedsQL-Rheumatology 3.0 module as the gold standard. We used two methods to assess correspondence between the two standardized measures: 1) Spearman’s correlation, and 2) the method of Bland and Altman [27]. The latter method evaluates the distribution of the differences and plots them against the standardized mean of the two measures.

Discriminative validity was evaluated by comparing median PRQL scores between subjects with physician-determined inactive disease (physician VAS of 0) versus active disease (physician VAS≥1), using a Wilcoxon rank sum test.

#### Responsiveness to change

Responsiveness of the PRQL to clinical change over time was tested in subjects with a follow-up visit 3–7 months after the initial study visit. We compared the mean change in PRQL between visits in subjects who had an improvement or worsening in disease activity according to the treating physician using a two-tailed t-test and the standardized response mean (SRM). The SRM is the mean PRQL score divided by the standard deviation of that score change [28]. SRMs were adjusted for the correlation between the 2 PRQL measurements in order to correctly apply Cohen’s thresholds of responsiveness [29]. An SRM ≥0.50 and ≥0.80 are considered evidence of moderate and large responsiveness, respectively [28,29].

## Results

### Sample characteristics

During the 10-month period we enrolled 161 children with JIA. Demographic and clinical characteristics are presented in Table 1. The most common JIA subtypes were persistent oligoarticular (N = 44, 27%) and RF-negative polyarticular (N = 36, 22%). Of those with available results 59 (37%) were ANA-positive, 7 (4%) were RF-positive, and 17 (11%) were HLA-B27 positive. This cohort had relatively low disease activity as measured by the physician VAS and subject/parent global health status VAS, and only 27% of subjects had active disease according to the ACR provisional criteria [21].

**Table 1:** Demographics

	**N (%)**
Age at visit, years (median, IQR)	11.6 (7.2, 15.5)
Disease duration, years (median, IQR)	2.8 (1.2, 5.5)
Male	43 (27)
Race	
White	132 (82)
Black	12 (8)
Asian	5 (3)
Pacific Islander	2 (1)
More than 1 race	1 (1)
Other	7 (4)
Unknown	2 (1)
ILAR category	
Systemic	14 (9)
Oligoarticular, persistent	44 (27)
Oligoarticular, extended	12 (7)
Polyarticular, RF-negative	36 (22)
Polyarticular, RF-positive	4 (3)
PsA	14 (9)
ERA	22 (14)
Undifferentiated	15 (9)
Physician disease activity VAS (median, IQR)	0 (0,1)
Physician-defined status	
Active disease	43 (27)
Inactive disease, on medication < 6 months	26 (16)
Clinical remission while on medication	53 (34)
Clinical remission, off medication <12 months	17 (11)
Clinical remission, off medication >12 months	19 (12)
Subject/parent global health status VAS	1 (0, 3)
Subject/parent pain VAS	1 (0, 4)

Legend: IQR= interquartile range, RF= rheumatoid factor, PsA= psoriatic arthritis, ERA= Enthesitis-related arthritis.

### Administration

The PRQL was simple for subjects and parents to comprehend. Completion of the PRQL required less than 5 minutes. Scoring of the questionnaire was straightforward and took less than 1 minute.

### Descriptive statistics

All PRQL items, including their psychometric properties, are shown in Table 2. Missing data was minimal (close to 5%) and considered to be missing completely at random.

**Table 2:** Pediatric rheumatology quality of life scale

	**Mean**	**SD**	**Skewness**	**Non-zero responses**	**Item-total scale correlation**
				**N (%)**	
**Total score**	2.60	3.02	1.45	(97) 60.2	
**Physical health subscale**	1.55	1.76	1.41	(91) 56.5	0.85
Item 1: Been limited in taking care of him/herself, that is, eating, dressing, or washing him/herself?	0.09	0.31	3.55	(12) 7.5	0.32
Item 2: Been limited in walking one block or climbing one flight of stairs?	0.21	0.44	1.96	(28) 17.4	0.55
Item 3: Been limited in doing activities that take a lot of energy, such as running, playing soccer, or dancing?	0.40	0.63	1.46	(49) 30.4	0.66
Item 4: Been limited in doing schoolwork or activities with friends?	0.17	0.40	2.06	(24) 17.9	0.68
Item 5: Had bodily discomfort?	0.69	0.70	0.86	(85) 52.8	0.68
**Psychosocial health subscale**	1.12	1.81	1.78	(60) 37.3	0.85
Item 6: Felt sad or blue?	0.27	0.50	1.65	(36) 22.4	0.71
Item 7: Felt anxious or acted nervous?	0.26	0.49	1.61	(35) 21.7	0.69
Item 8: Had troubles getting along with other children?	0.09	0.31	3.60	(12) 7.5	0.39
Item 9: Had difficulty concentrating or paying attention?	0.26	0.60	2.55	(28) 17.4	0.64
Item 10: Felt dissatisfied about his/her looks or abilities?	0.24	0.58	2.69	(26) 16.2	0.69

Legend: Each item was prefaced by the phrase: “Considering the part 4 weeks, how often has your child…”.

### Internal consistency reliability

Cronbach’s α was 0.81, 0.72, and 0.78 for the total score, PhH subscale and PsH subscale, demonstrating good internal consistency. Cronbach’s α for the PRQL total score and subscales among the following respondent subgroups were equal to or greater than 0.70: males, females, inactive disease, and active disease.

### Test-retest reliability

Forty-three enrolled subjects or parents were emailed a copy of the PRQL one week after the initial administration of the questionnaire. The average time between questionnaire completions was 11 days (IQR: 7,15). The pairwise correlation coefficient and ICC for test-retest reliability were 0.72 and 0.68, respectively, thereby demonstrating adequate reliability.

### Factor analysis

**Figure 1:**
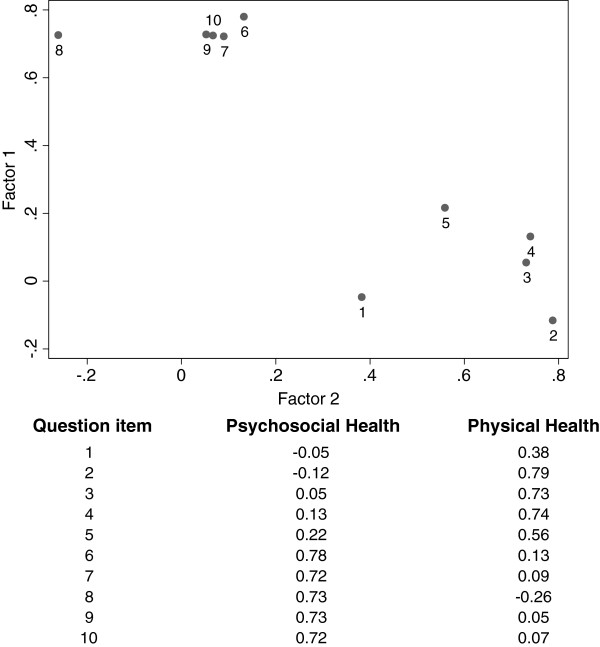
**Factor identification.** Questions 1–10 (see Table 2) of the PRQL loaded on two factors after promax rotation. Questions 1–5 have significant loadings (>0.30) on the factor “psychosocial health,” whereas questions 6–10 have significant loadings on the factor “physical health.”

A two-factor model adequately fit the data (CFI = 0.93; NNI = 0.91; RMSEA = 0.075). Post-hoc exploratory factor analysis, to check if a two-factor model would suitably fit the data if no assumptions were made about the data, provided further support for the two-factor structure (Figure 1).

### Convergent validity

**Figure 2:**
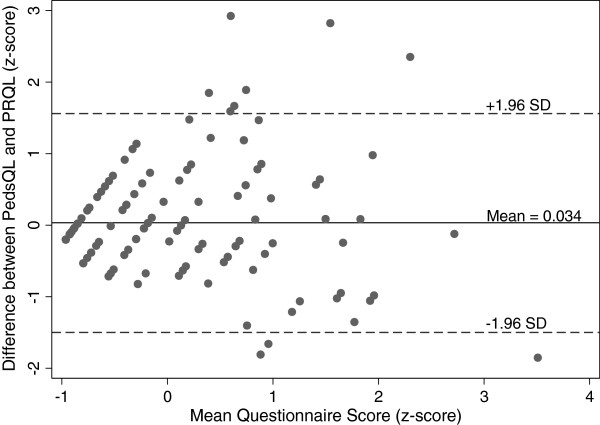
**Bland-Altman plot showing correspondence between PedsQL and PRQL.** Differences that fall within 1.96 standard deviations of the mean indicate substantial agreement and evidence of convergent validity.

We used two methods to assess correspondence between the PRQL and an established measure of HRQoL in JIA, the PedsQL generic core scale and the PedsQL rheumatology module: 1) Spearman’s correlation, and 2) the method of Bland and Altman [27]. The total PRQL score was highly correlated with the total PedsQL generic score and moderately correlated with 4 out of 5 of the PedsQL Rheumatology items (Table 3). The PRQL subscales PhH and PsH were also moderately correlated with the total PedsQL. The PhH correlated with the PedsQL Rheumatology items better than the PsH. Substantial agreement and evidence of convergent validity was also demonstrated using the Bland and Altman method of agreement since the great majority of differences fall within 1.96 standard deviations of the mean (Figure 2).

**Table 3:** Correlation between PRQL scores and the PedsQL generic and rheumatology modules and other clinical variable

	**PRQL score**		
	**Total**	**PhH**	**PsH**
**PedsQL**			
Generic core scales			
Total score	0.75^*^	0.65^+^	0.62^+^
Psychosocial health	0.66^+^	0.51^+^	0.65^+^
Physical health	0.71^*^	0.71^*^	0.45^+^
Rheumatology module			
Pain and hurt	0.69^+^	0.73^*^	0.41^+^
Daily activities	0.52^+^	0.52^+^	0.37
Treatment	0.34	0.34	0.24
Worry	0.44^+^	0.43^+^	0.35
Communication	0.42^+^	0.42^+^	0.30
**Parent/patient global health status VAS**	0.60^+^	0.66^+^	0.32
**Parent/patient pain VAS**	0.62^+^	0.67^+^	0.35
**Active joint count**	0.13	0.15	0.11
**Physician disease activity VAS**	0.28	0.31	0.12

Legend: *high correlation defined as r>0.7; ^+^Moderate correlation defined as 0.4>r<0.7; Poor correlation defined as ≤0.4.

We also assessed the correspondence between the PRQL and physician-determined disease activity and other PROs (Table 3). The PRQL and PhH subscale were moderately correlated with the parent’s assessment of global health status and pain. Correlations of the PRQL, PhH, and PsH and the active joint count and physician-determined disease activity were low. Correlations of the PedsQL total, physical health, and psychosocial health scores and the physician-determined disease activity were similarly low (0.32, 0.37, and 0.21, respectively).

### Discriminative validity

Discriminative validity was evaluated using physician disease activity VAS as the external criterion. Children with a physician disease activity VAS of 0 (N=89) and those with a VAS equal to or greater than 1 (N=57) were compared using a Wilcoxon rank sum test. The median PRQL scores for children with a physician VAS of 0 and ≥1 were 1 (IQR: 1,3) and 3 (IQR: 1,5), respectively (p-value <0.001).

### Responsiveness

**Figure 3:**
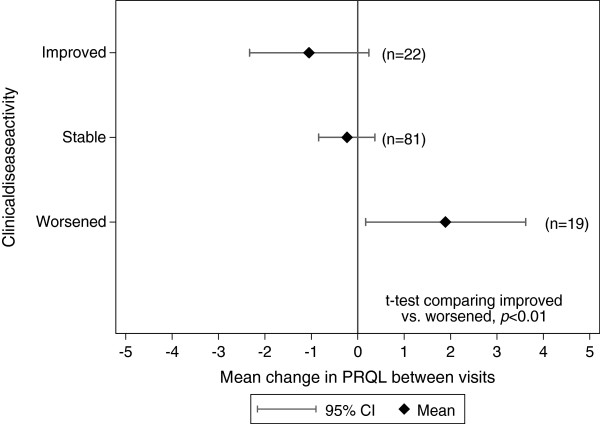
**Responsiveness of PRQL.** The difference in mean changes in PRQL scores between those who had improved and worsened disease activity were statistically significant by two-tailed t-test (*p*<0.01).

One hundred thirty one subjects had a PRQL recorded at a follow-up visit a median of 4.5 months (IQR: 3.0 to 6.4) after the baseline visit. Ninety subjects had stable disease activity, 22 improved, and 19 were worse according to the treating physician. Using the t-test the mean changes in PRQL scores were significantly different between those who improved and worsened according to physician assessment (p<0.01), demonstrating good responsiveness (Figure 3). The PRQL total score SRMs for those subjects who improved and worsened were 0.30 and 0.58 demonstrating small and moderate responsiveness, respectively.

## Discussion

The PRQL is a concise 10-item assessment of HRQoL that was created primarily for use in clinical practice. This prospective study supports the validity of this HRQoL assessment in JIA patients in our US clinical practice. Using cross-sectional data from 161 JIA subjects we have shown evidence of feasibility, convergent validity, discriminative validity, internal consistency, test-retest ability, and responsiveness. Content validity of the English PRQL was established prior to this study. The Italian PRQL was found to have strong content and face validity [13]. The Italian version was carefully forward- and back-translated to preserve the content of the instrument and observe any difficulties or problems with the translation. The rigorousness of the translation and testing of the translated version provide support for the content validity of the English version of the PRQL. Interestingly the correlations between the PRQL and the active joint count and physician disease activity VAS were low. Correlation between the PedsQL and physician disease activity VAS was similarly low. While these measurements assess different constructs of disease, one might expect that active disease greatly affects HRQoL, albeit not in a linear fashion. These findings highlight the importance of collecting PROs both in clinical practice and registries as they provide different qualitative information.

The brevity of this assessment tool makes it a feasible and non-onerous measure to collect and score as part of routine clinical practice. The limited number of items does not cover the breadth of other HRQoL assessments available for use in pediatric rheumatology including the PedsQL and JAQQ. However, we have shown moderate correlation with at least one of these measures, the PedsQL, by two different methods. For clinicians who aspire to collect PROs but do not have the luxury of time to administer, score, or pay for the PedsQL and/or JAQQ the PRQL is a reasonable alternative.

Our findings should be interpreted in the context of several limitations. First the validity assessment was confined to JIA patients and did not include other rheumatologic conditions. The validity of this measurement tool in other pediatric rheumatology conditions should be explored in future analyses. Second, our study population was a convenience sample of JIA subjects; however, they were recruited without regard to disease duration, disease severity, current disease activity, or therapy. Disease attributes may have differed in children who were and were not enrolled in this study; however, we expect that any selection bias would be non-differential in regards to this validity analysis.

## Conclusions

Despite caveats, our study indicates the PRQL is a valid tool for the assessment of HRQoL in children and adolescents with JIA in our US practice. Further it is easy to administer, simple and quick to complete, and takes minimal time and effort to score. This tool provides a relatively easy method to integrate PROs into routine clinical assessment and research.

## Competing interests

The authors declared that they have no competing interests.

## Authors’ contributions

PW was involved in study conception and design, data acquisition, analysis, and interpretation. AK was involved in data acquisition, analysis, and interpretation. JF was involved in data analysis and interpretation. CF was involved in study design and data interpretation. All authors were involved in manuscript preparation and have read and approved the final manuscript.

## References

[B1] Calvert M Blazeby J Altman DG Revicki DA Moher D Brundage MD Reporting of patient-reported outcomes in randomized trials: the CONSORT PRO extension JAMA 2013 309 8 814 822 10.1001/jama.2013.879 23443445

[B2] Yang X Xiao N Yan J The PedsQL in pediatric cerebral palsy: reliability and validity of the Chinese version pediatric quality of life inventory 4.0 generic core scales and 3.0 cerebral palsy module Qual Life Res 2011 20 2 243 252 10.1007/s11136-010-9751-0 20882356

[B3] Limbers CA Ripperger-Suhler J Heffer RW Varni JW Patient-Reported Pediatric Quality of Life Inventory 4.0 Generic Core Scales in Pediatric Patients with Attention-Deficit/Hyperactivity Disorder and Comorbid Psychiatric Disorders: Feasibility, Reliability, and Validity Value Health 2011 14 4 521 530 10.1016/j.jval.2010.10.031 21315637

[B4] Tsuji N Kakee N Ishida Y Validation of the Japanese version of the Pediatric Quality of Life Inventory (PedsQL) cancer module Health Qual Life Outcomes 2011 9 1 22 10.1186/1477-7525-9-22 21477361 PMC3096891

[B5] Varni JW Seid M Kurtin PS PedsQL 4.0: reliability and validity of the Pediatric Quality of Life Inventory version 4.0 generic core scales in healthy and patient populations Med Care 2001 39 8 800 812 10.1097/00005650-200108000-00006 11468499

[B6] Varni JW Seid M Smith Knight T Burwinkle T Brown J Szer IS The PedsQL in pediatric rheumatology: reliability, validity, and responsiveness of the pediatric quality of life inventory generic core scales and rheumatology module Arthritis Rheum 2002 46 3 714 725 10.1002/art.10095 11920407

[B7] PedsQL conditions of use: fees 2011 http://www.pedsql.org/conditions.html. Accessed May 13, 2011

[B8] Gao Y Johnston RC Karam M Pediatric sports-related lower extremity fractures: hospital length of stay and charges: what is the role of the primary payer? Iowa Orthop J 2010 30 115 118 21045983 PMC2958282

[B9] Dempsey RL Layde PM Laud PW Guse CE Hargarten SW Incidence of sports and recreation related injuries resulting in hospitalization in Wisconsin in 2000 Inj Prev 2005 11 2 91 96 10.1136/ip.2004.006205 15805437 PMC1730208

[B10] Fournie B Margarit-Coll N Champetier de Ribes TL Extrasynovial ultrasound abnormalities in the psoriatic finger. Prospective comparative power-doppler study versus rheumatoid arthritis Joint Bone Spine 2006 73 5 527 531 10.1016/j.jbspin.2006.01.019 16942893

[B11] Stoll ML Punaro M Psoriatic juvenile idiopathic arthritis: a tale of two subgroups Curr Opin Rheumatol 2011 23 5 437 443 10.1097/BOR.0b013e328348b278 21709556

[B12] Kuikka PI Pihlajamaki HK Mattila VM Knee injuries related to sports in young adult males during military service - Incidence and risk factors Scand J Med Sci Sports 2013 23 3 281 287 10.1111/j.1600-0838.2011.01397.x 22092849

[B13] Filocamo G Schiappapietra B Bertamino M A new short and simple health-related quality of life measurement for paediatric rheumatic diseases: initial validation in juvenile idiopathic arthritis Rheumatology (Oxford) 2010 49 7 1272 1280 10.1093/rheumatology/keq065 20338888

[B14] Consolaro A Ruperto N Filocamo G Seeking insights into the EPidemiology, treatment and Outcome of Childhood Arthritis through a multinational collaborative effort: introduction of the EPOCA study Pediatr Rheumatol 2012 10 39 10.1186/1546-0096-10-39 PMC3551702 23164467

[B15] Petty RE Southwood TR Manners P International League of Associations for Rheumatology classification of juvenile idiopathic arthritis: second revision, Edmonton, 2001 J Rheumatol 2004 31 2 390 392 14760812

[B16] Price DD Bush FM Long S Harkins SW A comparison of pain measurement characteristics of mechanical visual analogue and simple numerical rating scales Pain 1994 56 2 217 226 10.1016/0304-3959(94)90097-3 8008411

[B17] Carlsson AM Assessment of chronic pain. I. Aspects of the reliability and validity of the visual analogue scale Pain 1983 16 1 87 101 10.1016/0304-3959(83)90088-X 6602967

[B18] Bijur PE Latimer CT Gallagher EJ Validation of a verbally administered numerical rating scale of acute pain for use in the emergency department Acad Emerg Med 2003 10 4 390 392 10.1111/j.1553-2712.2003.tb01355.x 12670856

[B19] Singh G Athreya BH Fries JF Goldsmith DP Measurement of health status in children with juvenile rheumatoid arthritis Arthritis Rheum 1994 37 12 1761 1769 10.1002/art.1780371209 7986222

[B20] Ruperto N Ravelli A Pistorio A Cross-cultural adaptation and psychometric evaluation of the Childhood Health Assessment Questionnaire (CHAQ) and the Child Health Questionnaire (CHQ) in 32 countries. Review of the general methodology Clin Exp Rheumatol 2001 19 4 Suppl 23 S1 S9 11510308

[B21] Wallace CA Giannini EH Huang B Itert L Ruperto N American College of Rheumatology provisional criteria for defining clinical inactive disease in select categories of juvenile idiopathic arthritis Arthritis Care Res (Hoboken) 2011 63 7 929 936 10.1002/acr.20497 21717596

[B22] Harris PA Taylor R Robert T Payne J Gonzalz N Conde JG Research electronic data capture (REDCap) - a metadata-driven methodology and workflow process for providing translational research informatics support J Biomed Informatics 2009 42 2 377 381 10.1016/j.jbi.2008.08.010 PMC2700030 18929686

[B23] Streiner DL Norman GR Health measurement scales: a practical guide to their development and use 2003 3 Oxford; New York: Oxford University Press

[B24] Boers M Brooks P Strand CV Tugwell P The OMERACT filter for outcome measures in rheumatology J Rheumatol 1998 25 2 198 199 9489805

[B25] Gorsuch R Factor Analysis 1983 Hillsdale, New Jersey: Lawrence Erlbaum Associates, Inc, Publishers

[B26] Hatcher L A Step-by-Step Approach to Using SAS for Factor Analysis and Structural Equation Modeling 1994 1 Cary, NC: SAS Institute

[B27] Bland JM Altman DG Statistical methods for assessing agreement between two methods of clinical measurement Lancet 1986 1 8476 307 310 2868172

[B28] McGonagle D Ash Z Dickie L McDermott M Aydin SZ The early phase of psoriatic arthritis Ann Rheum Dis 2011 70 Suppl 1 i71 i76 10.1136/ard.2010.144097 21339224

[B29] McGonagle DG Helliwell P Veale D Enthesitis in psoriatic disease Dermatology 2012 225 2 100 109 10.1159/000341536 23108016

